# Internal hernia after laparoscopic-assisted proximal gastrectomy with jejunal interposition for gastric cancer: a case report

**DOI:** 10.1186/s40792-015-0051-3

**Published:** 2015-06-16

**Authors:** Kotaro Hirashima, Takashi Ishikawa, Shin-ichi Kosugi, Yosuke Kano, Yu Sato, Hiroshi Ichikawa, Takaaki Hanyu, Takeo Bamba, Toshifumi Wakai

**Affiliations:** Division of Digestive and General Surgery, Graduate School of Medical and Dental Sciences, Niigata University, 1-757 Asahimachi-dori, Chu-o-ku, Niigata, 951-8510 Japan

**Keywords:** Internal hernia, Laparoscopic-assisted proximal gastrectomy, Jejunal interposition

## Abstract

Internal hernia after gastrectomy is a rare complication. It can progress rapidly to vascular disturbance, necrosis, and perforation, therefore early diagnosis and surgical treatment is essential. We present a case of internal hernia following laparoscopic-assisted proximal gastrectomy with jejunal interposition reconstruction in a 68-year-old man, who presented with acute abdominal pain and vomiting. Computed tomography showed a whirl sign, ascites, and a closed-loop formation of the small intestine. We diagnosed an internal hernia and performed emergency surgery. Laparotomy revealed chyle-like ascites and extensive small intestine with poor color. We recognized that about 20 cm of jejunum from the ligament of Treitz was strangulated behind the pedicle of the jejunum lifted during laparoscopic-assisted proximal gastrectomy. We relieved the strangulation, whereupon the color of the strangulated intestine was restored. Therefore, we did not perform intestinal resection and reconstruction. Finally, we fixed the jejunal pedicle and mesentery of the transverse colon. We report this case as there are few reported cases of internal hernia after laparoscopic-assisted proximal gastrectomy.

## Background

Laparoscopic gastrectomy for gastric cancer is one of the common treatments for patients with early gastric cancer, not only in Asia but also in Western countries, with numerous clinical series being reported [1–3]. Internal hernia is a potential complication known to occur after surgery, which can lead to closed-loop bowel obstruction. Patients with internal hernia may present with nonspecific and intermittent abdominal pain, nausea, vomiting, and abdominal distension.

One of the forms of internal hernia is Petersen’s hernia, which is an internal hernia that can occur through a mesenteric defect caused by Roux-en Y anastomosis (RY). Use of the RY reconstruction during distal and total gastrectomy has been gradually increasing in recent years, most likely to avoid anastomotic leakage and reflux inflammation [4, 5]. Furthermore, RY reconstruction is often used following laparoscopic Roux-en-Y bypass (RYGBP) which is the most commonly performed bariatric surgery. However, it has been suggested that because it induces fewer postoperative adhesions, the laparoscopic approach leads to an increased risk of internal hernia. For several years, internal hernia has been recognized as a potential surgical complication of RYGBP, with an incidence of 5.0 to 9.0 % [6, 7]. We have performed jejunal interposition (JI) for proximal gastrectomy in recent years. As the reason, in several reconstruction methods including JI, esophagogastrostomy (EG), jejunal pouch interposition (JPI), and double tract reconstruction (DT), we consider considering that JI is the most physiological anastomosis because of maintaining normal digestive function and oncological radicality [8, 9]. Both RY reconstruction and JI reconstruction lift the jejunum in a similar manner, so it is possible that the risk of internal hernia exists at a similar level in both operations.

This case of internal hernia was formed by passing behind the jejunal pedicle at the side of the laparoscopic-assisted proximal gastrectomy (LAPG). There are few reported cases of internal hernia after LAPG, so we report this case as our experience with internal hernia since the introduction of LAPG with retrocolic JI reconstruction.

## Case presentation

**Fig. 1:**
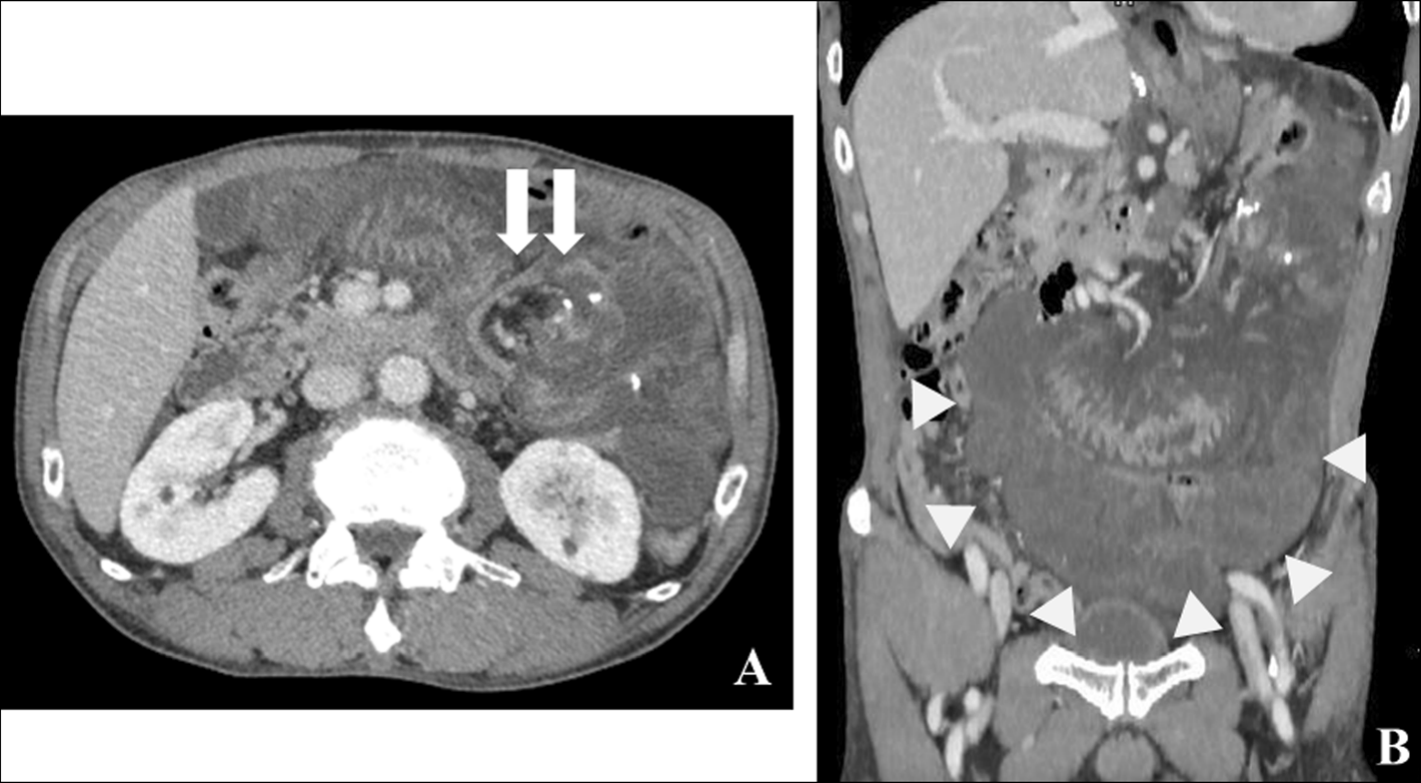
Abdominal CT scan showing whirl sign (*white arrows*), ascites, and closed-loop formation of the small intestine (*white arrowheads*): **a** axial section; **b** coronal section

A 68-year-old man presented with sudden abdominal pain and vomiting. He had no other symptoms. He underwent LAPG for early gastric cancer 2 years earlier. Laboratory test results were almost normal, including those for tumor markers (i.e., carcinoembryonic antigen (CEA), cancer antigen (CA) 19-9) (Table 1). Results of computed tomography (CT) of the abdomen revealed a beak sign, ascites, and a closed-loop formation of the small intestine (Fig. 1). There was no evidence of distant metastasis or intra-abdominal lymphadenopathy, and the other organs appeared normal. We diagnosed internal hernia and performed emergency surgery.

**Table 1:** Laboratory data on admission

WBC	11480/μl	Na	142 mEq/l
RBC	428 × 104/μl	K	3.3 mEq/l
Hb	13.5 g/dl	Cl	107 mEq/l
Hct	39.9 %	Ca	9.9 mEq/l
Plt	29.3 × 104/μl	Cr	0.68 mg/dl
		BUN	24 mg/dl
AST	18 U/l		
ALT	12 U/l	PT	86 %
T-Bil	0.8 mg/dl	PT-INR	1.07
D-Bil	0.1 mg/dl	APTT	83.8 %
ALP	296 U/l		
γ-GTP	19 U/l	CEA	6.1 ng/dl
TP	7.1 g/dl	CA19-9	35 U/ml
Alb	4.7 g/dl		
LDH	181 U/l		
CRP	0.02 mg/dl

**Fig. 2:**
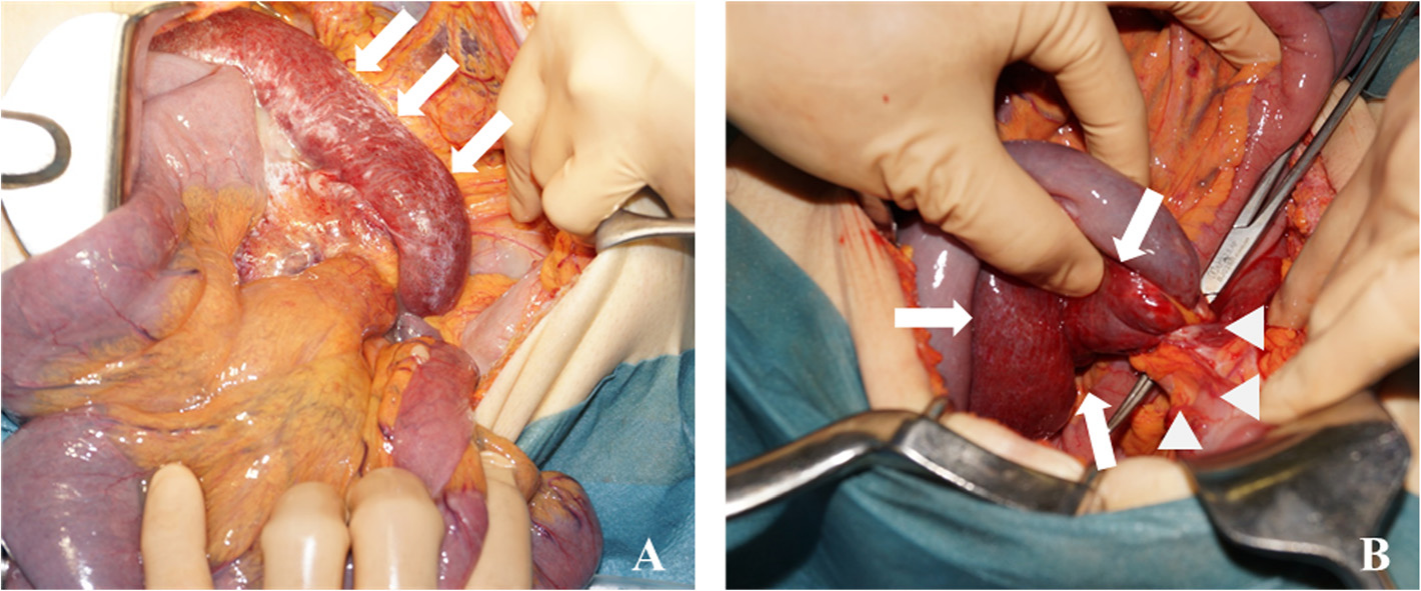
**a** Laparotomy showing chyle-like ascites and extensively damaged small intestine (*white arrows*). **b** About 20 cm of the jejunum from the ligament of Treitz (*white arrows*) was strangulated behind the lifting jejunal pedicle (*white arrowheads*) of the jejunal interposition formed during laparoscopic-assisted proximal gastrectomy

**Fig. 3:**
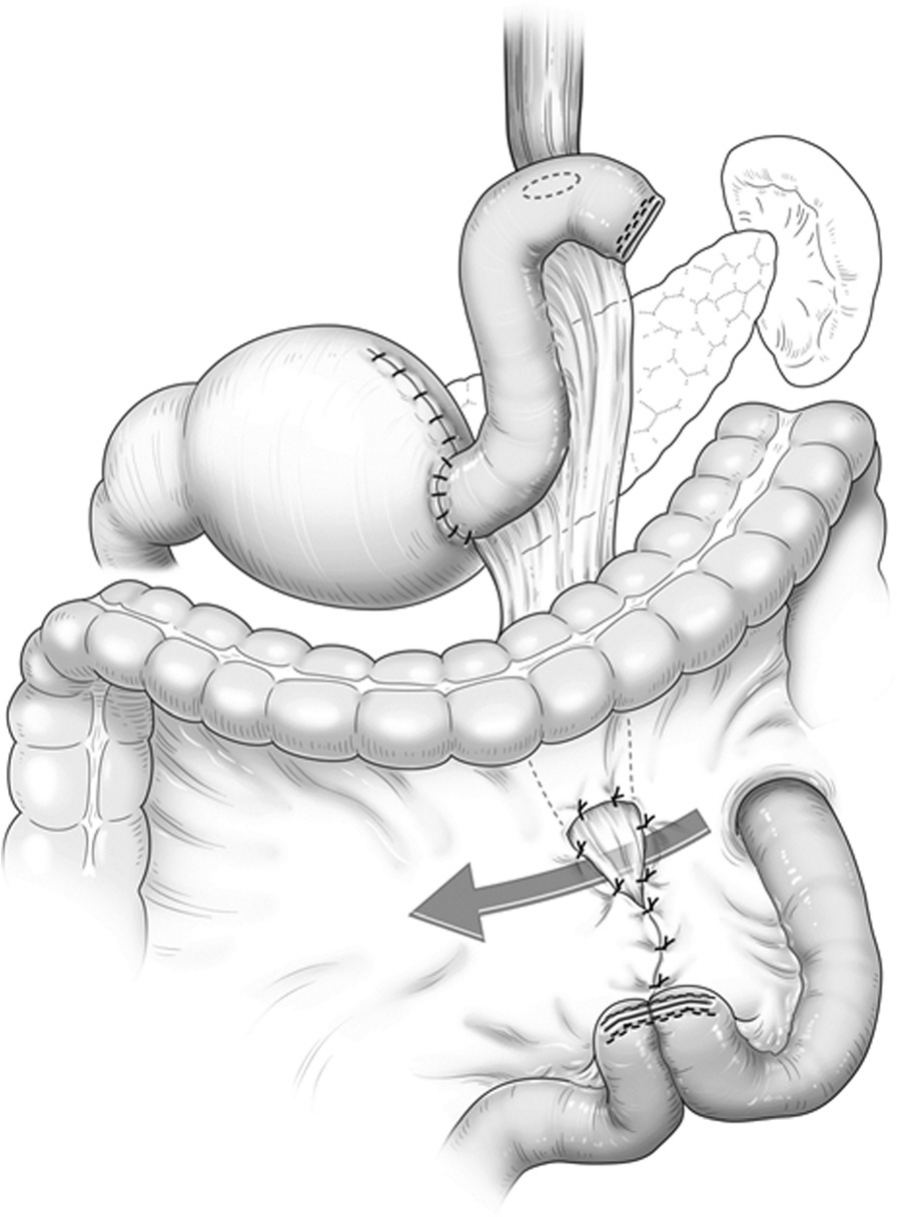
Schema showing detailed findings of the internal hernia in this case

We performed laparotomy, whereupon we discovered chyle-like ascites and extensive discoloration of the small intestine. We recognized that about 20 cm of jejunum from the ligament of Treitz was strangulated behind the jejunal pedicle lifted following JI at LAPG (Figs. 2 and 3). The leading part contained about 30 cm of jejunum including the jejunojejunostomy formed during the LAPG. We relieved the strangulation; whereupon, the color of the strangulated intestine was restored immediately, so intestinal resection and reconstruction were not performed. Finally, we fixed the lifting jejunal pedicle and mesentery of the transverse colon to prevent recurrence.

The postoperative progress was initially good, with the patient starting to take a meal on the fourth postoperative day. However, he gradually developed abdominal pain and vomiting, which was managed with fasting and fluids. As abdominal CT did not reveal the reason for obstruction, we performed intestinal endoscopy. Although it passed into the area surrounding the jejunojejunostomy, it was very difficult to pass the flexure at this site. We considered that improvement from continuing conservative treatment was unlikely and so we re-operated on the 23rd postoperative day. Laparotomy revealed that the 20 cm of jejunum from the ligament of Treitz, including the jejunojejunostomy, had adhered at the point of fixation of the lifted jejunal pedicle and the mesentery of transverse colon. We separated the adhesion of the jejunum and re-formed the jejunojejunostomy. After reoperation, the patient recovered uneventfully and was discharged from hospital on the 13th day post-secondary surgery.

### Discussion

Laparoscopic surgery is generally associated with several advantages over the open approach to the same surgery, including reduction in postoperative pain and earlier resumption of normal activities. Several randomized controlled studies of laparoscopic-assisted distal gastrectomy (LADG) show further benefits including reduced bleeding and improved quality of life when compared with a traditional open distal gastrectomy [2, 10, 11]. In patients with gastric cancer who have undergone LADG with RY reconstruction, there exists Petersen’s defect, which is the space between the Roux limb and the transverse mesentery. Thus, there is an increased probability of internal hernia after LADG with RY reconstruction [12]. In Japan, Billroth-I reconstruction has been traditionally performed after LADG [12, 13]. Therefore, the incidence of internal hernia after LADG in Japan is extremely low.

However, there are several reconstruction methods for proximal gastrectomy, such as JI, EG, JPI, and DT. In our institute, we have performed JI reconstruction following LAPG with a retrocolic approach for a long time. For the surgical procedure of LAPG, the stomach was almost half transected. After the jejunum was divided about 20 cm from the ligament of Treitz, the distal jejunum was placed posterior to the transverse colon. An end-to-side anastomosis was performed by using one 25-mm circular stapler for the esophagojejunostomy, and one 60-mm linear stapler was used for the jejunal stamp. We designed the length of free jejunum to be about 12 cm; an end-to-end anastomosis was performed for gastrojejunostomy. Finally, a side-to-side jejunojejunostomy was performed. We consider JI reconstruction to be the most physiological anastomosis, because the distal stomach remains to maintain normal digestive function, and the JI reconstruction has a similar role to the cardiac sphincter in reducing gastroesophageal reflux inflammation [8, 9]. JI reconstruction is also similar to RY reconstruction in that a short length of jejunum is lifted while keeping blood circulation via a retrocolic approach through the transverse mesentery. As in RY reconstruction, the jejunal pedicle is lifted through the transverse mesentery. Therefore, there is also the possibility of internal hernia after LAPG with JI reconstruction. We should verify and close the gap between the transverse mesentery and jejunal pedicle lifted during LAPG to prevent internal hernia.

## Conclusions

We experienced a case that was diagnosed as internal hernia after LAPG with JI reconstruction for gastric cancer. Internal hernia is a rare complication which, however, needs to be diagnosed quickly and treated with emergency surgery.

## Consent

Written informed consent was obtained from the patient for publication of this case report and any accompanying images. A copy of the written consent is available for review by the Editor-in-Chief of this journal.

## Abbreviations

RY: Roux-en-Y anastomosis
RYGBP: Roux-en Y bypass
JI: jejunal interposition
EG: esophagogastrostomy
JPI: jejunal pouch interposition
DT: double tract reconstruction
LAPG: laparoscopic-assisted proximal gastrectomy
CEA: carcinoembryonic antigen
CA: cancer antigen
CT: computed tomography
LADG: laparoscopic-assisted distal gastrectomy

## References

[1] Kitano S, Shiraishi N, Uyama I, Sugihara K, Tanigawa N (2007). Japanese laparoscopic surgery study group. A multicenter study on oncologic outcome of laparoscopic gastrectomy for early cancer in Japan. Ann Surg.

[2] Kim HH, Hyung WJ, Cho GS, Kim MC, Han SU, Kim W (2010). Morbidity and mortality of laparoscopic gastrectomy versus open gastrectomy for gastric cancer: an interim report–a phase III multicenter, prospective, randomized trial (KLASS Trial). Ann Surg..

[3] Guzman EA, Pigazzi A, Lee B, Soriano PA, Nelson RA, Benjamin Paz I (2009). Totally laparoscopic gastric resection with extended lymphadenectomy for gastric adenocarcinoma. Ann Surg Oncol..

[4] Comeau E, Gagner M, Inabnet WB, Herron DM, Quinn TM, Pomp A (2005). Symptomatic internal hernias after laparoscopic bariatric surgery. Surg Endosc..

[5] Hosoya Y, Lefor A, Ui T, Haruta H, Kurashina K, Saito S (2011). Internal hernia after laparoscopic gastric resection with antecolic Roux-en-Y reconstruction for gastric cancer. Surg Endosc..

[6] Himpens J, Verbrugghe A, Cadiere GB, Everaerts W, Greve JW (2012). Long-term results of laparoscopic Roux-en-Y Gastric bypass: evaluation after 9 years. Obes Surg..

[7] Aghajani E, Jacobsen HJ, Nergaard BJ, Hedenbro JL, Leifson BG, Gislason H (2012). Internal hernia after gastric bypass: a new and simplified technique for laparoscopic primary closure of the mesenteric defects. J Gastrointest Surg..

[8] Zhao P, Xiao SM, Tang LC, Ding Z, Zhou X, Chen XD (2014). Proximal gastrectomy with jejunal interposition and TGRY anastomosis for proximal gastric cancer. World J Gastroenterol..

[9] Nozaki I, Hato S, Kobatake T, Ohta K, Kubo Y, Kurita A (2013). Long-term outcome after proximal gastrectomy with jejunal interposition for gastric cancer compared with total gastrectomy. World J Surg..

[10] Kodera Y, Fujiwara M, Ohashi N, Nakayama G, Koike M, Morita S (2010). Laparoscopic surgery for gastric cancer: a collective review with meta-analysis of randomized trials. J Am Coll Surg..

[11] Kim YW, Baik YH, Yun YH, Nam BH, Kim DH, Choi IJ (2008). Improved quality of life outcomes after laparoscopy-assisted distal gastrectomy for early gastric cancer: results of a prospective randomized clinical trial. Ann Surg..

[12] Kojima K, Inokuchi M, Kato K, Motoyama K, Sugihara K (2014). Petersen's hernia after laparoscopic distal gastrectomy with Roux-en-Y reconstruction for gastric cancer. Gastric Cancer..

[13] Takaori K, Nomura E, Mabuchi H, Lee SW, Agui T, Miyamoto Y (2005). A secure technique of intracorporeal Roux-Y reconstruction after laparoscopic distal gastrectomy. Am J Surg..

